# Wettability Investigations and Wet Transfer Enhancement of Large-Area CVD-Graphene on Aluminum Nitride

**DOI:** 10.3390/nano7080226

**Published:** 2017-08-18

**Authors:** Marius Knapp, René Hoffmann, Volker Cimalla, Oliver Ambacher

**Affiliations:** 1 Fraunhofer Institute for Applied Solid State Physics IAF, Tullastraße 72, 79108 Freiburg, Germany; rene.hoffmann@iaf.fraunhofer.de (R.H.); volker.cimalla@iaf.fraunhofer.de (V.C.); 2 Department of Power Electronics, Institute of Sustainable Systems Engineering (INATECH), University of Freiburg, 79085 Freiburg, Germany; oliver.ambacher@inatech.uni-freiburg.de

**Keywords:** graphene wet transfer, aluminum nitride, wettability, chemical vapor deposition, surface plasma treatment, graphene doping, CVD-graphene

## Abstract

The two-dimensional and virtually massless character of graphene attracts great interest for radio frequency devices, such as surface and bulk acoustic wave resonators. Due to its good electric conductivity, graphene might be an alternative as a virtually massless electrode by improving resonator performance regarding mass-loading effects*.* We report on an optimization of the commonly used wet transfer technique for large-area graphene, grown via chemical vapor deposition, onto aluminum nitride (AlN), which is mainly used as an active, piezoelectric material for acoustic devices. Today, graphene wet transfer is well-engineered for silicon dioxide (SiO_2_). Investigations on AlN substrates reveal highly different surface properties compared to SiO_2_ regarding wettability, which strongly influences the quality of transferred graphene monolayers. Both physical and chemical effects of a plasma treatment of AlN surfaces change wettability and avoid large-scale cracks in the transferred graphene sheet during desiccation. Spatially-resolved Raman spectroscopy reveals a strong strain and doping dependence on AlN plasma pretreatments correlating with the electrical conductivity of graphene. In our work, we achieved transferred crack-free large-area (40 × 40 mm^2^) graphene monolayers with sheet resistances down to 350 Ω/sq. These achievements make graphene more powerful as an eco-friendly and cheaper replacement for conventional electrode materials used in radio frequency resonator devices.

## 1. Introduction

Graphene is a monolayer of hexagonal-oriented carbon atoms with outstanding electronic properties [1]. Its high electrical conductivity combined with its virtually massless character makes graphene interesting for electrodes in the field of acoustic devices operated at radio frequency (RF), such as surface and bulk acoustic wave resonators. Aluminum nitride (AlN) is a typically used material for such RF resonator devices [2,3]. Several groups as [4,5] could show that graphene reveals its potential as a conductive material to minimize the influence on the performance of resonator devices. For an unfailing use as an active electrode, graphene needs to exhibit low sheet resistances since the conductivity of the electrode material strongly affects the quality factor of a resonator [6,7]. These findings require high-quality large-area graphene on insulating substrates. Chemical vapor deposition (CVD) is the most promising technique for the synthesis of large-area graphene layers with a low sheet concentration of structural defects. Since the synthesis occurs on catalytic substrates [8], such as copper (Cu) and nickel (Ni), graphene needs to be transferred on substrates used for applications in electronic devices. In order to ensure a large-area continuous graphene sheet on the target substrate the transfer procedure within the graphene process, technology must be stable, avoiding the formation of broken bonds and cracks which decrease the electric performance of graphene. The standard transfer technique is wet transfer on silicon dioxide (SiO_2_) substrates [9,10]. Sheet resistances *R_S_* down to 1.1 kΩ/sq are achieved reproducibly [11]. Transferred graphene with *R_S_* = 450 Ω/sq is commercially available from certain suppliers [12], which proves the mature process technology und usability of CVD graphene. In the field of RF microelectromechanical systems (RF MEMS), used e.g., in wireless communication, graphene is able to reveal both its conductive and two-dimensional character as an alternative electrode material in full strength. In this case, the transfer substrates are of piezoelectric nature, such as aluminum nitride (AlN). Graphene transfer on AlN is already adopted by many groups [4,13,14], but a thorough examination and experimental optimization of the transfer method has not yet been shown. However, this is essential in order to improve the structural as well as the electric quality of graphene on the transfer substrate.

In this publication, we show an optimized wet transfer technique on AlN. As a two-dimensional conductive material, graphene can play an important role as an alternative electrode material regarding the elimination of viscous losses due to its virtually massless character. Using the wet transfer technique [15], the desiccation of graphene on the target substrate is the most critical process step. The interplay between baking after the process and the substrate’s wettability strongly influences this desiccation procedure. In our work we found that hydrophobic surfaces tend to be responsible for additional strain-induced cracks in transferred graphene sheets. Therefore, the target surface has a great impact on the success of the wet transfer.

Commonly used silicon dioxide (SiO_2_) as a target substrate is highly hydrophilic, therefore, no other surface treatment than standard substrate cleaning is necessary for a successful transfer [10]. However, the RF topic requires AlN, which is a relatively hydrophobic surface substrate, due to its piezoelectric character for RF resonator devices. The emerging cracks in the graphene monolayer result in very high sheet resistances and strongly decrease the performance of graphene as a conducting electrode material. Therefore, a surface plasma pretreatment process was developed to modify the surface energy of AlN. The increased wettability enables a more controlled and smooth desiccation and, concurrently, lower graphene sheet resistance. Our obtained results ensure an unchanged graphene layer quality during both synthesis and transfer and are therefore fundamental for ongoing research activities in the field of acoustic RF devices [16] using graphene as an active electrode on AlN. Depending on the plasma there is both a physical and a chemical effect on the surface properties which influences both the transfer and the performance of the fabricated device. The surface roughness of the substrate surface strongly influences the adhesion between graphene and substrate. The adhesion, represented by a van der Waals interaction, depends on the surface roughness due to a changing mean distance between graphene and the substrate, as Gao et al. showed [17]. Additionally, the chemical effect acts like a “cleaning” of the surface [18]. Our investigations show that using the chemical cleaning effect of plasma pretreatments to optimize the transfer process is beneficial. As a result, we are able to achieve large-area graphene transferred on AlN with *R_S_* ≈ 350 Ω/sq as a best value, which is strongly comparable to commercially available graphene on SiO_2_.

## 2. Results

### 2.1. Wettability Investigations on AlN/Si Substrates

**Figure 1:**
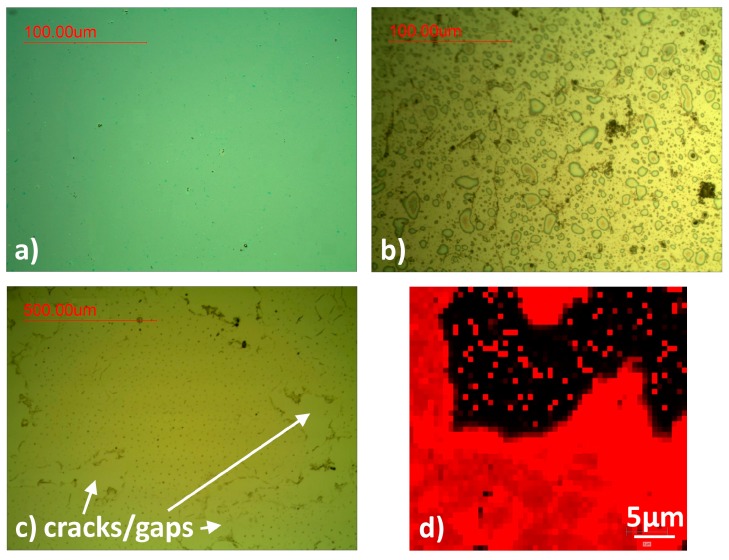
(**a**) Graphene on SiO_2_. (**b**,**c**) Graphene on untreated aluminum nitride (AlN). Circular structures show aqueous cavities (**b**). Arrows indicate areas without graphene (**c**,**d**) Raman 2D peak mapping of a randomly selected area with cracks/gaps (absent 2D peak, black) clearly visible and break the graphene layer (red).

**Figure 2:**
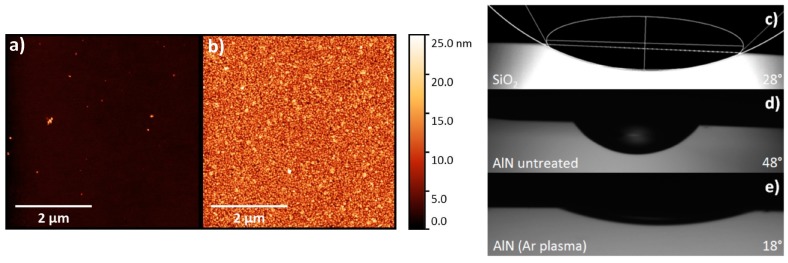
Atomic force microscopy (AFM ) image of thermally oxidized SiO_2_ on Si (**a**) and AFM image of AlN sputtered on Si (**b**). (**c**–**e**) Optical images of a water droplet on different substrates—contact angles are obtained (**d**) via a circular and elliptical fit.

**Figure 3:**
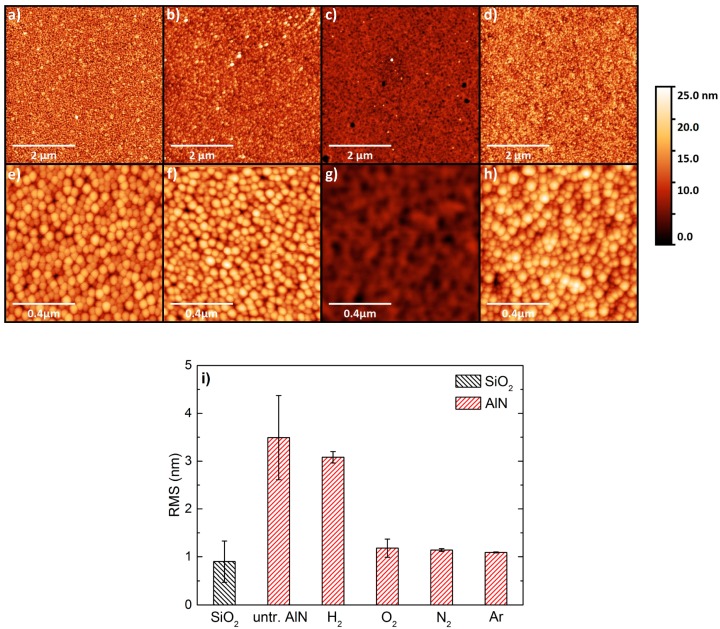
(**a**–**h**) AFM images of differently plasma pretreated AlN with different enlargement. As grown AlN on Si (**a**,**e**), H_2_ plasma treatment (**b**,**f**) and Ar plasma treatment (**c**,**g**). An adjusted plasma process for of AlN on Bragg mirror structures (AlN-SMR), named as B2 in Section 2.2, reveals that the typically columnar structure of the AlN surface is preserved, which is shown exemplary in (**d**,**h**). (**i**) Comparison of RMS surface roughness parameter for SiO_2_ (black) and differently plasma treated AlN/Si surfaces.

Graphene transferred on an untreated AlN/Si substrate is shown in Figure 1. Optical microscope images (Figure 1b,c) indicate the cracks and gaps in the transferred graphene layer which stem from the desiccation process. The characteristic Raman 2D peak clearly reveals large graphene-free areas (Figure 1d). In contrast, graphene on SiO_2_ does not show cracks after transfer under the same transfer conditions (a). In order to investigate surface effects, SiO_2_ on Si and AlN on Si were characterized via atomic force microscopy (AFM). Figure 2 clearly shows that the SiO_2_ surface (a) is explicitly smoother than the AlN surface (b). The calculated root mean square (RMS) as a surface roughness parameter averages between 0.9 ± 0.43 nm and 3.49 ± 0.88 nm for SiO_2_ and AlN, respectively (Figure 3i). The much lower roughness for SiO_2_ might be a first indication that the graphene transfer is less stable on the rougher AlN surface. 

Since the wettability of the surface depends both on surface roughness and chemical conditioning, contact angle (CA) measurements were performed. According to the sessile drop method [19] very small water droplets of 10 µL volume were used for the measurements, so that additional mass effects influencing the droplet shape can be neglected. Therefore, the contact angle can be obtained via a circular fit of the optical droplet image (Figure 2c). For untreated SiO_2_ and AlN on Si the contact angle is 28° and 48°, respectively. Both AFM and CA measurement clearly reveal the different surface properties resulting in an unsatisfying graphene transfer on AlN. In a next step, AlN/Si samples were plasma treated in different ways before the transfer. The samples were directly exposed to H_2_, N_2_, O_2_ and Ar plasmas. Figure 3 shows the related AFM images obtained for each AlN sample. In Figure 3, (a) shows an image of untreated AlN, (b) illustrates AlN after an H_2_ plasma, and (c), AlN after an Ar plasma. Figure 3e–g show a zoom-in to the corresponding samples. Ar plasma treatment significantly changes the topography of AlN whereas there is almost no change for H_2_ plasma pretreatment. The effect of N_2_ and O_2_ (not shown) is fairly similar as the one for Ar. Comparing the corresponding roughness parameters we obtained RMS values for N_2_, O_2_ and Ar (1.1–1.2 nm), which are close to the one for SiO_2_ (0.9 nm) (Figure 3i). For the H_2_ plasma the RMS value merely changes to 3.2 nm. 

**Figure 4:**
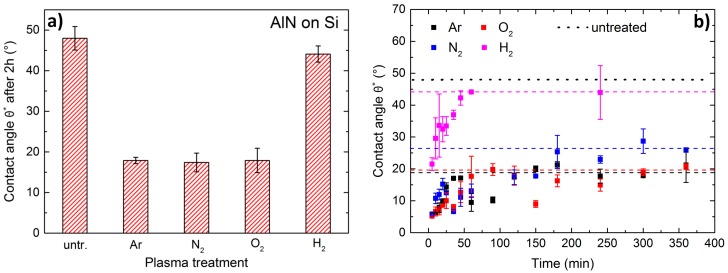
(**a**) Contact angle (CA) for all pretreatment variations measured after 2 h. (**b**) Time-dependent contact angle measurements for Ar, O_2_, N_2_ and H_2_ plasma pretreatment of AlN on Si. The black dotted line represents CA for untreated AlN on Si. A recovery effect is strongly visible.

Corresponding CA measurements show a strong change in surface properties for the differently treated AlN samples. The obtained CA (Figure 4a) for measurements 2 h after pretreatment closely fit the RMS values. CA tremendously decreases to approx. 18° for N_2_, O_2_ and Ar plasma pretreatments. In general, the contact angle between a fluid and a solid is described by the Young equation [20]:

(1) $$\gamma_{SA}-\gamma_{SL}-\gamma_{LA}\times \cos\theta =0$$

with the surface tension γ and the corresponding indices S (solid), A (air) and L (liquid) for the phases. θ is called the Young or chemical angle which describes the contact angle for a perfectly flat surface. According to the Wenzel model which describes homogenous wetting regimes, surface roughness changes the contact angle via

(2) $$\cos\theta^{*}=r\times \cos\theta$$

where $\theta^{*}$ is the corrected contact angle including the roughness parameter *r*, which is the ratio between the real and the projected solid surface area [21]. The Wenzel model establishes the connection of surface roughness on surface wettability. Higher roughness leads to lower wettability. Heavy atoms like N_2_, O_2_ and Ar physically damage the AlN structure and smoothen its surface, whereas H_2_ has almost no major physical impact on AlN due to its low atomic weight. The surface damage is clearly seen in Figure 3g. The typically pebble-like structure for sputtered AlN is destroyed, although the surface is smoother than without any plasma treatment.

Apart from this physical smoothing which influences the substrates wettability, there is another important effect responsible for the surface properties. Plasma treatment also leads to a change in the chemical composition of the surface. For all plasmas there is a cleaning effect of the surface. Contamination atoms like carbon and other carbonic composites, which tend to be hydrophobic, are both physically and chemically removed. These composites can readsorb on the AlN surface after the plasma pretreatment [22], which indicates a time-dependent recovery effect of the AlN substrate’s wettability.

In addition to this plasma cleaning effect, Sönmez et al. [18] explore another impact of different plasma gases on stainless steel. In case of an O_2_ plasma, there is an oxidation effect of the surface which in turn becomes more hydrophilic due to a smoothening. A certain amount of residual O_2_ within the reactor chamber might enable oxidizing reactions which results also in a smoothening of the AlN surface. A nanolayer of aluminum oxide allotropes directly increases the wettability. Depending on the fabricated application one needs to keep in mind that oxidation might cause undesired side effects on the device performance (see Section 2.2). Dehydration effects, e.g., during the wet transfer, might decrease the wettability again. 

Physical damage in combination with N_2_ or H_2_ plasma might also form nitrate composites, such as Al(NO_3_)_3_ and NH_4_NO_3_, due to the aforementioned, non-negligible amount of residual oxygen in the plasma chamber. Despite their negligible effect in physical surface smoothening, H_2_ atoms might create labile hydroxide compounds with the assistance of background oxygen, such as Al(OH)_3_. These chemical reactions can also activate the AlN surface due to the availability of aluminum atoms in the substrate material. The creation of nitrate components as intermediate products of chemical reactions increase, as well as oxides, the surface energy i.e., wettability [22]. Nitrate components are unstable and tend to degrade very soon and also dehydration takes place right after the plasma treatment. On the contrary an oxidized layer on the AlN surface can endure. All of these described effects can strongly influence the total surface wettability and might explain the recovery effect below. 

The “cleaning” phenomenon of the removal of surface contaminations can be seen in Figure 4b. Time-dependent CA measurements obviously show a recovery effect. It is clearly visible that there is a strong reduction in θ* for all applied plasmas within the first minutes after plasma exposure. This drop in CA is rapidly recovered within one hour after pretreatment. The recovery effect is quickest for H_2_ plasma. There is a specific saturation CA value for each of the plasma pretreatments, which is time-independent and averages between 44° for H_2_ and 18° for N_2_, O_2_ and Ar (dashed lines). Hence these maximum values of $\theta^{*}$ for *t* > 2 h for different plasmas represent the ones shown in Figure 4a. There exists an explicitly noticeable hydrophobic recovery with increasing time. By repeating the plasma pretreatment, the time-dependent change of the contact angles can be obtained at every measurement cycle. The difference in recovery might be explained with the above mentioned chemical reactions. For Ar, N_2_, and O_2_ plasma several oxidation reactions superpose the cleaning effects, whereas for H_2_ plasma a stable oxidation apparently does not take place. For this purpose a profound analysis of the chemical compounds on the AlN surface after plasma treatment needs to be carried out, this is not within the scope of this publication.

In a first summary we can conclude that plasma pretreatments impact the surface both physically and chemically, whereas a chemically activated surface shows an aging effect. Transfer experiments indicate that already a physical impact on the AlN surface resulting in as little as θ* < 20° improves the quality of transferred graphene. The Raman D/G ratio decreases below 0.2 and the crack density can be lowered significantly. As shown later on this influences directly the graphene sheet resistance.

### 2.2. Wettability Investigations on BAW-SMR Substrates

**Figure 5:**
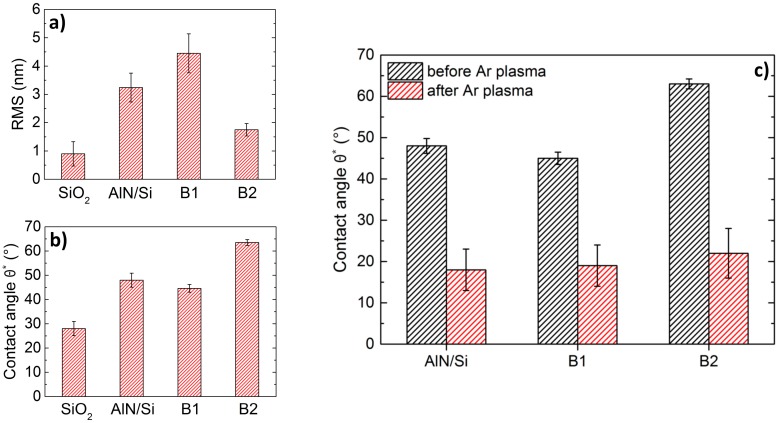
(**a**) Comparison of roughness values (RMS) obtained from AFM images for AlN/Si, B1 (AlN/TiAlCuW) and B2 (AlN/MoAlN) substrates, SiO_2_ as a reference. (**b**) Comparison of corresponding CA obtained by dropping water droplets on the surface. (**c**) Comparison of CA before and 30 min after Ar plasma treatment.

Since we are interested in graphene as a top electrode for resonator devices, we adapted the transfer process including the plasma treatment on solidly mounted bulk acoustic wave AlN resonator structures (BAW-SMR, described in detail in Section 4.1) B1 and B2, which were grown on a Bragg reflector structure and different bottom electrodes. In this case, an increase in wettability without a change in surface roughness and stable oxide allotropes is preferable, to avoid a potentially different performance characteristic of the resonator devices due to a changed AlN surface roughness. This is important regarding the performance characteristics of actual devices, such as BAW resonators, where a structural change of the surface can influence future device functionality. For this reason we adjusted the plasma treatment in a further experimental step by reducing power and ambient pressure during the plasma process to lower the direct plasma exposure of the AlN substrates. In this case the change in wettability occurs only because of the chemical conditioning of the surface and not as a consequence of physical damage of the AlN surface. Monolayer graphene was therefore transferred on AlN grown on a material stack working as a solidly mounted resonator device (BAW-SMR). Figure 5a,b show RMS obtained from AFM images and CA values, respectively, for both resonator structures. SiO_2_ and AlN on Si are shown as a reference. Although the two samples differ in their surface roughness (RMS = 4.4 ± 0.7 nm (1.8 ± 0.2 nm) for B1 (B2)) the CA is remarkably high for B2 (63 ± 6°). When an Ar plasma is applied on both samples RMS values do not change (AFM image in Figure 3d,h), but CA strongly decreases to 19–22° (Figure 5c). The comparably large error bars result from a relatively fast recovery effect (CA measurements are performed within 30 min after plasma treatment). Hence both a physical damage and stable oxidation effects can be ruled out. These experimental results demonstrate that wettability manipulations of the AlN surface are possible without physical surface smoothening which might deteriorate other device properties. For this, the transfer step needs to occur directly after the plasma pretreatment.

### 2.3. Strain and Doping Investigations via Spatially-Resolved Raman Spectroscopy

**Figure 6:**
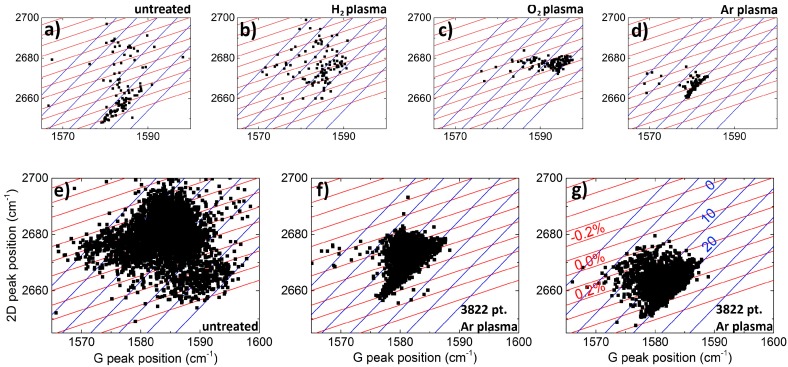
Raman spectroscopy measurements of graphene on AlN—2D peak position (ω_2*D*_) versus G peak position (ω*_G_*). Line scan (121 data points) for differently pretreated AlN/Si substrates (**a**–**d**). Raman map of graphene on AlN (3822 data points, 30 × 50 µm^2^ area) for untreated AlN/Si (**e**), Ar plasma pretreated AlN/Si substrate (**f**) and Ar plasma pretreated B1 substrate (**g**). Shift in doping visible due to addition of AuCl_3_ during etching process. Level curves for compressive and tensile strain (blue line, in %) and for doping concentration *n* (red line, in 10^12^ cm^−2^).

Additionally, the strain distribution across the transferred graphene layer was investigated to further understand the effect of plasma pretreatment. Using spatial Raman spectroscopy analysis of the 2D and G peak positions, ω_2*D*_ and ω*_G_*, respectively, one is able to draw conclusions about strain and charge carrier density due to the strain and doping sensitivity of the Raman G and 2D modes [23]. In Figure 6, ω_2*D*_ versus ω*_G_* is plotted for graphene on differently pretreated AlN substrates. The red (blue) line shows different levels of hole doping (strain). The slopes are obtained from [23], as

(3) $${\left(\frac{\Delta \omega_{2D}}{\Delta \omega_{G}}\right)}_{\varepsilon }\;=2.2\pm 0.2,$$

(4) $${\left(\frac{\Delta \omega_{2D}}{\Delta \omega_{G}}\right)}_{n}^{hole}=0.70\pm 0.05.$$

According to the authors, electron doping would show a nonlinear slope and in this case Equation (4) would not hold. Hall measurements of graphene on AlN showed hole doping, which allows the use of the aforementioned equations. Das et al. [24,25] revealed a ω_2*D*_ dependency on hole doping as $\Delta \omega_{2D}/\Delta n=-1.04\;$ cm, *n* in terms of 10^12^ cm^−2^. Yoon et al. [26] calculate the dependence of strain on a shift of ω*_G_* as $\Delta \omega_{G}/\Delta \varepsilon =-23.5\;$ cm^−1^/%. These two relations lead to a direct identification of strain and doping of graphene on the differently pretreated AlN substrates.

In Figure 6, a Raman line scan of 121 data points is plotted (a–d). ω_2*D*_ versus ω*_G_* reveals a significant change for plasma pretreated AlN on Si. Strain and doping levels are labelled in Figure 6g and hold for each of the graphs. A rather linear dependency is visible for both O_2_ and Ar plasmas, whereas there is no dependency identifiable for untreated AlN and AlN pretreated with an H_2_ plasma. Graphene on Ar (O_2_) plasma pretreated AlN shows dominantly tensile strain between 0.1–0.3% (0.1–0.2%); the doping is significantly lower for the Ar plasma (*n* = 2 × 10^11^–1 × 10^13^ cm^−1^) compared to the O_2_ plasma (*n* = 2 × 10^13^–4 × 10^13^ cm^−1^). The same result is obtained for N_2_ (not shown). We can conclude that variations of strain and doping are strongly reduced applying an N_2_, O_2_ or Ar plasma instead of an H_2_ or no plasma pretreatment at all. To obtain a greater spatial distribution map measurements were done, shown in (e–g) for untreated and Ar plasma treated AlN substrate (AlN/Si and B1). Due to observable D peak intensities for graphene on untreated AlN (e) information about actual doping concentrations cannot be extracted due to overlapping effects in the Raman shifts. However, a clear reduction of strain variation is apparent for the Ar plasma for both AlN/Si and B1 (f,g). The same results hold for B2 (not shown). This indicates that the transfer-induced strain results not from surface roughness itself but from wettability effects on the graphene sheet during transfer. For the transfer of graphene on B1 we added exemplary AuCl_3_ during the Cu etching, which results in an additional doping of the transferred layer. This is directly visible in a shift of the 2D/G ratio (g). AuCl_3_ might play an important role regarding the decrease of sheet resistance due to the doping effect. This will be investigated further.

## 3. Discussion

**Figure 7:**
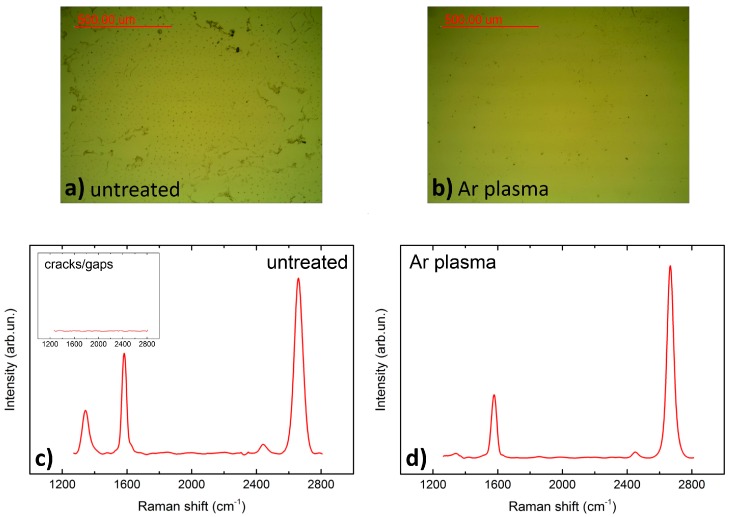
Comparison of transferred graphene on untreated AlN (**a**,**c**) and Ar plasma treated AlN (**b**,**d**). Optical microscope images show avoidance of cracks/gaps in the graphene sheet for Ar plasma treated AlN (**b**). Raman spectroscopy image shows an average of 121 measurements (line scan); inset in (**c**) shows no graphene Raman signal in gap areas. D peak is strongly reduced for Ar plasma treated AlN substrate (**d**).

In order to quantify the enhanced graphene quality due to the applied plasma treatments the samples were investigated via optical microscopy and Raman spectroscopy. Figure 7 shows an obvious improvement of the graphene wet transfer. Cracks and large-area gaps are avoided and the graphene sheet is transferred completely (microscopic images). Raman spectroscopy shows a clear quality enhancement. The defect-related D peak (1350 cm^−1^) vanishes almost completely, whereas the 2D/G intensity ratio increases up to 2.6. The 2D peak lies at 2772 cm^−1^. Both indicate the existence of mostly monolayer graphene on AlN [27]. This result directly corresponds with the reduction of strain variation after Ar plasma treatment (Section 2.3). Non-uniform in-plane strain in graphene raises the probability of creating cracks and wrinkles in the layer.

**Figure 8:**
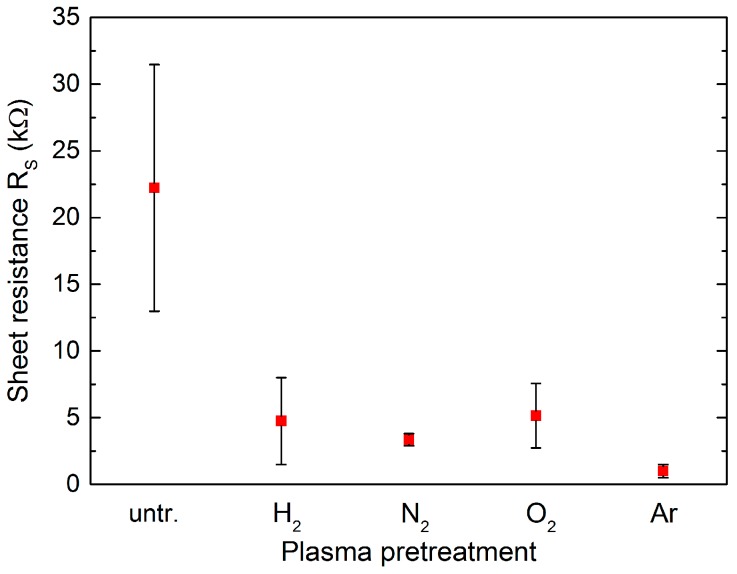
Sheet resistance of graphene monolayers transferred on AlN/Si as a comparison for different surface plasma pretreatment. Relatively high measurement errors obtained for untreated AlN due to 1 mm probe distance of the measurement setup.

The obtained quality improvement is directly proven via four-point measurements. Due to plasma pretreatment, the sheet resistance *R_S_*, shown in Figure 8, drops almost one order of magnitude for all applied plasmas (from 22 kΩ/sq to <5 kΩ/sq). The high error bars, especially for the untreated AlN samples, result from the probe distance of 1 mm, which makes resistance measurements strongly dependent on the created gaps. According to Raman measurements, small graphene domains might have slightly lower sheet resistances. The most reliable results are achieved for Ar plasma pretreatment. Here, *R_S_* constantly reaches values below 1 kΩ/sq. Best values were obtained as *R_S_* = 350 Ω/sq. Sheet resistance measurements were homogenous on an area of 40 × 40 mm^2^. So far, we can exclude the effect of induced additional strain due to doping, as shown in Figure 6g. Therefore, a further reduction of *R_S_* seems to be promising. Additional experiments on this topic still need to be carried out.

**Figure 9:**
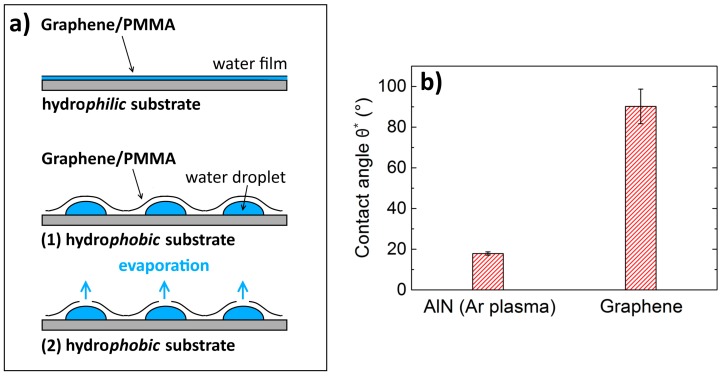
(**a**) Schematic figure of the wettability concept. Different wettability for hydrophobic and hydrophilic target surfaces influence the desiccation process resulting in gaps/cracks and non-uniform strain within the transferred graphene layer. (**b**) Comparison of CA for Ar-treated AlN and a pure graphene surface.

As a conclusion, our experiments clearly reveal that the most delicate process step of the wet transfer technique is the graphene’s desiccation on the target substrate. Figure 9a shows the wettability concept on different substrates. Due to their high wettability, hydrophilic surfaces (such as SiO_2_ (1)) ensure a continuous desiccation via capillary forces in between the substrate and the graphene/PMMA (Poly (methyl methacrylate)) whereas hydrophobic surfaces (such as AlN (2)) form water droplets instead. These droplets either remain in between the substrate and the graphene layer (1) or they break the graphene layer for sublimation, creating cracks and discontinuities (2). As shown experimentally in this work, additional strain is applied on the transferred graphene sheets, which critically influences the sheet resistance *R_S_* of the graphene. The underlying effect of hydrophobic surfaces is the concept of surface and interfacial energies, which we identified indirectly via CA measurements. Hydrophobic surfaces feature a low surface energy and hydrophilic surfaces feature a high surface energy [28]. Because of the high surface energy of water due to its polar character, the surface energy of AlN must be increased. In our experiments, this was done by plasma treatment. Thinking of a further optimization of graphene as an electrode material, a stacking of several graphene sheets transferred on top of each other might be reasonable to further decrease *R_S_*. Problems regarding the transfer of graphene onto graphene will occur due to its extremely hydrophobic behavior. CA measurements revealed θ* to be very close to 90° (Figure 9b). This means that the topic of surface wettability will continue to arise with respect to the fabrication of high-quality large-area graphene multilayers. Furthermore, an area-increase of transferred graphene towards wafer-size makes the desiccation process even more challenging.

## 4. Materials and Methods

### 4.1. Materials

In this work, the synthesis of a graphene monolayer was achieved via chemical vapor deposition (CVD), a mature and commonly applied technique of growing graphene [29], on copper (Cu) foil (25 µm thickness, 99.8% purity, Alfa Aesar, Haverhill, MA, USA) used as a catalytic metal substrate in a cold wall low-pressure reactor (BlackMagic Pro, Aixtron, Herzogenrath, Germany) using methane and hydrogen as precursor and carrier gas, respectively (flow ratio CH_4_/H_2_ = 25%, 1600 Pa, 980 °C, 30 min deposition time).

Primarily, AlN substrates were investigated which are fabricated via sputtering (2 µm AlN on 500 µm silicon (AlN/Si, 4″ wafer size)). The AlN wafers were cut into 20 × 20 mm^2^ pieces for 10 × 15 mm^2^ graphene transfers. Solidly mounted BAW resonators (BAW-SMR) with sputtered AlN as a piezoelectric layer (3″ wafer size, 1.7 µm AlN on TiAlCuW (named B1) and 1.5 µm AlN on MoAlN (B2)) were used for 40 × 40 mm^2^ graphene transfers (Table 1). Both AlN/Si and AlN-SMR wafers were fabricated by our project partner within the Graphene Flagship and provided to us for transfer experiments.

### 4.2. Methods

The graphene on Cu foil was routinely investigated by a Raman spectroscopy line scan of 121 data points (InVia Raman microscope, 532 nm excitation laser, grating 1800 lines/mm, Renishaw, Gloucestershire, UK), since it is an approved, easily applicable, non-destructive characterization method [30]. The representative 2D/G peak intensity ratio of reproducibly above 2.5 and the FWHM (Full Width at a half Maximum) of the 2D peak of <30 cm^−1^ clearly identifies this as a monolayer structure [27,31]. An almost imperceptible defect peak (D/G ratio < 0.15) supports the conclusion that our synthesized material is a low-defect monolayer of graphene.

**Figure 10:**
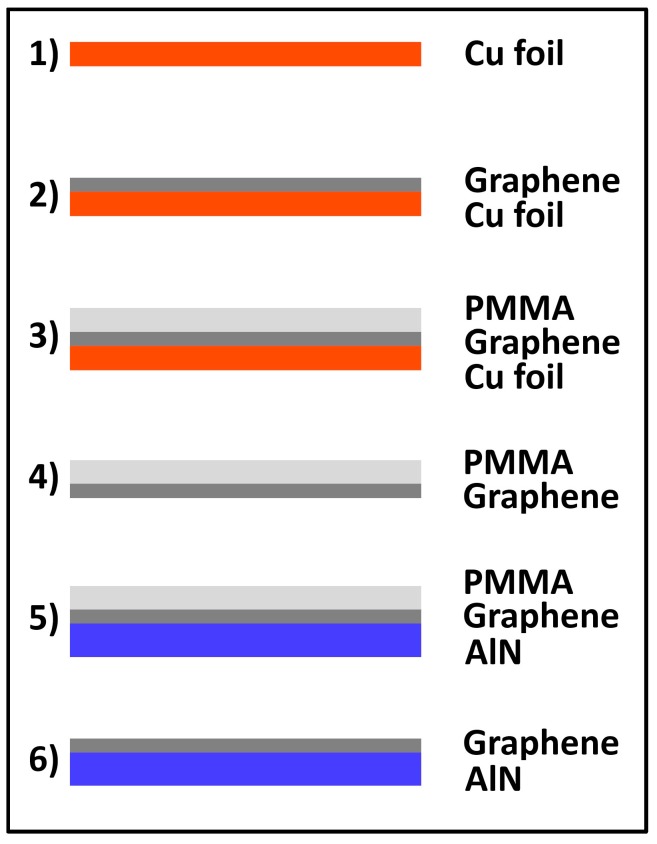
Schematic illustration of standard wet transfer process on aluminum nitride (AlN) after graphene chemical vapor deposition (CVD) growth.

After CVD synthesis, the graphene monolayers were transferred on differently pretreated AlN substrates using the wet transfer technique (Figure 10), described in detail in [15], in a slightly aligned procedure. Using a PMMA (Poly (methyl methacrylate)) coating as a protection layer of the graphene/Cu stack the copper foil was etched off with an aqueous ammonium persulfate solution ((NH_4_)_2_S_2_O_8_, 0.5 mol/L). This turned out to result in a cleaner interface between AlN and graphene after transfer due to lower formation of precipitates and chemical byproducts than commonly used etching solvents like iron chloride (FeCl_3_) and iron nitrate (Fe(NO_3_)_3_). Wu et al. [32] suggest (NH_4_)_2_S_2_O_8_ as an etching solvent since it avoids additionally induced Fe compounds during etching, which reduce the quality of the grown graphene. The PMMA/graphene stack was then deposited onto different AlN target substrates. Before the graphene transfer, the AlN substrates were exposed to different plasmas (H_2_, N_2_, O_2_ and Ar, 10 min) in order to vary the surface conditions regarding surface energy and roughness. The SiO_2_ substrates received no plasma treatment and were used as reference samples. For comparison purposes, graphene transferred on untreated AlN was also investigated. Plasma pretreatments of AlN samples were done in a direct current (DC) plasma reactor (Leybold, 100–300 W, 9 × 10^−3^–5 × 10^−2^ mbar, 10 min exposure time). The wettability of the AlN sample was characterized via contact angle measurements using the sessile drop method [33] in a self-construction experimental setup. Here, a precise test droplet of deionized (DI) water (10 µL) was dispensed on the AlN surface. The obtained video image of the droplet was fitted (ImageJ, image processing software, contact angle plugin) to extract the contact angle. The AlN surface roughness was investigated with atomic force microscopy (AFM; Nanowizard III, JPK instruments, Berlin, Germany). In addition, the sheet resistance of the transferred graphene was measured with the four-point method (Loresta-GX MCP-T700, 1 mm probe distance, Mitsubishi Chemical Analytech, Yamato, Japan).

## 5. Conclusions

The wet transfer of CVD graphene monolayers onto AlN was investigated. We identified a strong dependence of the wettability of AlN surfaces on different plasma pretreatments due to both a physical and a chemical effect on the AlN surface. AFM images and contact angle measurements reveal a change of surface properties resulting in spatially lower strain variation and a tremendous reduction of layer cracks and gaps for N_2_, O_2_ and Ar plasma pretreatments. Focusing on the chemical “cleaning” of the surface leads to a temporal but strong increase of wettability, which enables a defect-free graphene transfer without changing the AlN’s surface morphology. Sheet resistance measurements show a direct effect of this optimization regarding sheet conductivity. *R_S_* can reproducibly be reduced below 1 kΩ/sq for transferred graphene with the size of 40 × 40 mm^2^ and an Ar plasma pretreatment. 

With this new knowledge, the wet transfer method for the assembly of multilayer graphene sheets for further sheet resistance reduction should be investigated further. A sensitive adjustment of the surface properties of graphene regarding its strong hydrophobic behaviour is essential to obtain even lower sheet resistances. Furthermore, the effect of substrate surface pretreatment is an interesting and inevitable topic for the transfer of high quality graphene onto other substrate materials. Our work clearly shows a strong improvement in the quality of graphene and therefore emphasizes the promising future of graphene as a virtually massless and ultrathin electrode material for high-frequency devices.

## Figures and Tables

**Table 1:** Overview about used AlN samples for the graphene wet transfer.

Substrate	Substrate Size	Graphene Layer Size
AlN/Si	20 × 20 mm^2^	10 × 15 mm^2^
B1 (AlN/TiAlCu)	3″ wafer	40 × 40 mm^2^
B2 (AlN/MoAlN)	3″ wafer	40 × 40 mm^2^

## Abbreviations

In this manuscript the following abbreviations are used:

RF	Radio frequency
MEMS	Microelectromechanical system
CVD	Chemical vapor deposition
AlN	Aluminum nitride
PMMA	Poly (methyl methacrylate)
BAW	Bulk acoustic wave
SMR	Solidly mounted resonator
AFM	Atomic force microscopy
CA	Contact angle
